# Effects of the Missense Mutations in Canine BRCA2 on BRC Repeat 3 Functions and Comparative Analyses between Canine and Human BRC Repeat 3

**DOI:** 10.1371/journal.pone.0045833

**Published:** 2012-10-12

**Authors:** Yasunaga Yoshikawa, Kazuhiko Ochiai, Masami Morimatsu, Yu Suzuki, Seiichi Wada, Takahiro Taoda, Satomi Iwai, Seishiro Chikazawa, Koichi Orino, Kiyotaka Watanabe

**Affiliations:** 1 Laboratory of Veterinary Biochemistry, School of Veterinary Medicine, Kitasato University, Aomori, Japan; 2 Department of Basic Science, School of Veterinary Nursing and Technology, Nippon Veterinary and Life Science University, Tokyo, Japan; 3 Division of Disease Model Innovation, Institute for Genetic Medicine, Hokkaido University, Sapporo, Japan; 4 Laboratory of Veterinary Radiology and Radiation Biology, School of Veterinary Medicine, Kitasato University, Aomori, Japan; 5 Laboratory of Small Animal Surgery 2, School of Veterinary Medicine, Kitasato University, Aomori, Japan; 6 Laboratory of Small Animal Surgery 1, School of Veterinary Medicine, Kitasato University, Aomori, Japan; 7 Laboratory of Small Animal Internal Medicine 2, School of Veterinary Medicine, Kitasato University, Aomori, Japan; University of Nebraska Medical Center, United States of America

## Abstract

Mammary tumors are the most common tumor type in both human and canine females. Mutations in the breast cancer susceptibility gene, BRCA2, have been found in most cases of inherited human breast cancer. Similarly, the canine BRCA2 gene locus has been associated with mammary tumors in female dogs. However, deleterious mutations in canine BRCA2 have not been reported, thus far. The BRCA2 protein is involved in homologous recombination repair via its interaction with RAD51 recombinase, an interaction mediated by 8 BRC repeats. These repeats are 26-amino acid, conserved motifs in mammalian BRCA2. Previous structural analyses of cancer-associated mutations affecting the BRC repeats have shown that the weakening of RAD51's affinity for even 1 repeat is sufficient to increase breast cancer susceptibility. In this study, we focused on 2 previously reported canine BRCA2 mutations (T1425P and K1435R) in BRC repeat 3 (BRC3), derived from mammary tumor samples. These mutations affected the interaction of canine BRC3 with RAD51, and were considered deleterious. Two BRC3 mutations (K1440R and K1440E), reported in human breast cancer patients, occur at amino acids corresponding to those of the K1435R mutation in dogs. These mutations affected the interaction of canine BRC3 with RAD51, and may also be considered deleterious. The two BRC3 mutations and a substitution (T1430P), corresponding to T1425P in canine BRCA2, were examined for their effects on human BRC3 function and the results were compared between species. The corresponding mutations and the substitution showed similar results in both human and canine BRC3. Therefore, canine BRCA2 may be a good model for studying human breast cancer caused by BRCA2 mutations.

## Introduction

Mammary tumors are the most common tumor type in both human and canine females, and the molecular biological similarities between the tumors in both species are well conserved [1]–[4]. In humans, the breast cancer susceptibility gene, *BRCA2,* has been identified as a tumor-suppressor gene. Consequently, germ-line mutations in *BRCA2* predispose women to a high risk of breast cancer. Genetic analyses, including detection of deleterious mutations, to identify carriers of *BRCA2* mutation is strongly advocated as the lifetime risk of breast cancer is high (81–88%) for women carrying this mutation [5], [6].

The BRCA2 protein is involved in homologous recombination repair by its interaction with RAD51 recombinase, a process mediated by BRC repeats [7]. BRC repeats are conserved motifs in BRCA2, approximately 26 amino acids in length. All mammalian BRCA2 proteins contain 8 BRC repeats, clustered on exon 11, and located in the central portion of the protein; these repeats show significant sequence conservation [8]. These structural characteristics indicate the importance of the BRC domain.

In female dogs, mammary tumors comprise about half of the tumors typically observed, and approximately half of these mammary tumors are found to be malignant [9], [10]. No other animal has such a high incidence of mammary tumors. A recent study involving single nucleotide polymorphism analysis of intronic markers suggested that the canine *BRCA2* gene locus is associated with mammary tumors [11], [12]. Consistent with this notion, we have previously demonstrated that loss of heterozygosity, which is one of the mechanisms of *BRCA2* inactivation, in mammary tumors [13]. Previous studies have provided basic information on canine BRCA2, such as the consensus sequence and information regarding its function, including the interactions between the BRC repeats of canine BRCA2 and RAD51 [14]–[18]. Canine BRCA2 mutations, including 2 (T1425P and K1435R) in BRC repeat 3 (BRC3), have been identified as potential mutations related to tumorigenesis [13], [19], [20]. However, the effects of these mutations on the function of canine BRCA2 have not been identified, thus far.

In this study, 2 canine BRCA2 mutations, located in BRC3, were found to affect interactions with RAD51. Mutations (K1440R and K1440E) in the amino acid sequence corresponding to the K1435R mutation in canines have also been reported in patients with breast cancer. Canine mammary tumors are good models for human breast cancer, but the specific worth of canine BRCA2 has not been evaluated [21], [22]. Therefore, we confirmed the effects of these mutations in human BRC3 and a T1430P substitution corresponding to the T1425P mutation in canine BRCA2 on BRC3 function and compared between species. The findings suggest that canine BRCA2 may be a good model for studying human breast cancer caused by analogous mutations.

## Materials and Methods

### Crystal structure modeling

The crystal structure of the human RAD51-BRC4 complex was retrieved from the Research Collaboratory for Structural Bioinformatics Protein Data Bank (http://www.rcsb.org/(PDB ID: 1N0W)) and analyzed using the University of California, San Francisco (UCSF) Chimera software (http://www.cgl.ucsf.edu/chimera/) [23].

### Protein expression and purification

The glutathione-*S*-transferase (GST)-fused wild-type and mutant canine BRC3 (cBRC3) (aa 1410–1482) peptides were expressed in the pGEX6p-1 vector (GE Healthcare, Waukesha, WI, USA) in Rosetta II cells (Novagen, Madison, WI, USA) and purified using glutathione sepharose 4B (GE Healthcare). Mutations were introduced to the cBRC3 sequence by using an In-Fusion® HD Cloning Kit (TaKaRa, Tokyo, Japan).

### GST pull-down assay

In a total volume of 50 µl, 300 nM GST-fused peptides were incubated with 53.6 nM human RAD51 (hRAD51) (Bio Academia, Osaka, Japan) in 50 mM Tris (pH 8.0), 150 mM NaCl, 1 mM EDTA and 1 mM DTT (TBS buffer) at 4°C for 1 h. Glutathione sepharose 4B was equilibrated in TBS buffer containing 5% bovine serum albumin (BSA) and incubated with the protein–peptide complexes at 4°C for 1 h. Complexes bound to glutathione sepharose 4B were thoroughly washed in TBS buffer and suspended in sodium dodecyl sulfate-polyacrylamide gel electrophoresis (SDS-PAGE) loading buffer. The samples were analyzed by SDS-PAGE and western blotting with anti-RAD51 IgG (1∶1000; Santa Cruz Biotech, Santa Cruz, CA, USA) or anti-GST IgG (1∶500; Medical & Biology Laboratories, Aichi, Japan), and horseradish peroxidase conjugated anti-rabbit IgG (Cappel Laboratories, Cochranville, PA, USA). Blots were developed using the ECL Plus Western Blotting Detection System (GE Healthcare).

### Competitive ELISA

To test the interference abilities of the GST-cBRC3 peptides on the hRAD51-cBRC3 interactions, wild-type and mutant GST-cBRC3s were allowed to compete against the GST-cBRC3 peptide (in the solid phase) for recombinant full-length hRAD51 (Bio Academia). Ninety-six-well plates (Maxisorp, Nunc, Roskilde, Denmark) were coated overnight at 4°C with 5 µg/mL GST-cBRC3 peptide in TBS buffer. The plates were then washed with PBS containing 0.05% Tween 20 (PBS-T) and blocked with ELISA buffer (PBS containing 0.1% gelatin and 0.05% Tween 20) for 1 h at room temperature. After blocking, the plates were washed 3 times with PBS-T, and inhibitor peptides at the indicated concentrations in TBS buffer containing hRAD51 (1 µg/mL) were added in a total volume of 50 µL in the coated ELISA plates and incubated overnight at 4°C. The plates were then washed 3 times with PBS-T and incubated for 1 h at 37°C with the anti-RAD51 antibody H-92 (diluted 1∶5000 with ELISA buffer; Santa Cruz Biotech). The plates were washed 3 times with PBS-T and incubated for 1 h at 37°C with alkaline phosphatase (ALP)-labeled goat anti-rabbit IgG antibody (diluted 1∶5000 with ELISA buffer; Cappel Laboratories). The plates were then washed 3 times with PBS-T, following which *p*-nitrophenyl phosphate was added and the color was allowed to develop for about 30 min at 37°C; the reaction was stopped by adding 200 mM EDTA. Absorbance was measured at 405 nm using a VERSAmax Tunable Microplate Reader (Molecular Devices, Sunnyvale, CA, USA).

### Mammalian two-hybrid assay

For the mammalian cell two-hybrid assay, the coding regions of cBRC3, canine BRC4 (cBRC4) (GenBank ID: NM_001006653), human BRC3 (hBRC3) and BRC4 (hBRC4) (GenBank ID: NM_000059), canine RAD51 (cRAD51) (GenBank ID: NM_001003043), hRAD51 (NM_002875) and the N-terminus of cBRCA2 (aa 1–1000) were cloned into pM and pVP16 plasmids (Clontech, Mountain View, CA, USA). The method for generating the luciferase reporter plasmid, pGluc, and those for the mammalian two-hybrid assays were as described previously [24]. The binding interference assay was performed by the modified mammalian two-hybrid assay. VP16 and DNA-binding domain (DBD)-fused cRAD51 constructs were introduced into HeLa cells (Riken Cell Bank, Ibaraki, Japan) with interference constructs, namely, wild-type and mutant cBRC3 in NLS-pEGFP-C1 [15].

### Immunostaining and microscopy

HeLa cell monolayers (80% confluent) cultured on coverslips (Matsunami Glass, Osaka, Japan) were transfected with the NLS-pEGFP-C1 vectors containing wild-type or mutant cBRC3 by using FuGENE HD (Promega, Madison, WI, USA). After 24 h, irradiation of transfected HeLa cells was carried out in a Faxitron CP160 irradiator (Faxitron X-ray Corporation, Tucson, AZ, USA) at 5 Gy/min for a total dose of 15 Gy. The cells were immediately returned to the tissue culture incubator and were fixed with 10% formalin 18 h after irradiation. After permeabilization with 0.3% Triton X-100 in PBS, the cells were incubated with H-92, a polyclonal antibody against hRAD51, followed by Alexa Fluor-568-conjugated goat anti-rabbit IgG (Molecular Probes, Eugene, OR, USA). Cell nuclei were stained with Hoechst 33342 (Molecular Probes); hRAD51 foci were examined under a fluorescence microscope (Nikon Corporation, Tokyo, Japan).

### Statistical analysis

All statistical analyses were performed using an F test followed by Student's *t*-test or a one-way analysis of variance (ANOVA) followed by a Dunnett's or Tukey's test.

## Results

### Canine BRC3 strongly interacts with canine and human RAD51

**Figure 1:**
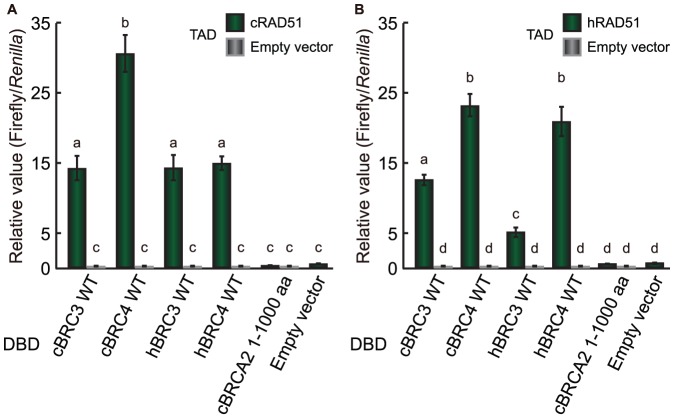
Interaction between cBRC3 and canine or human RAD51 detected by mammalian two-hybrid assay. The level of the interaction between cBRC3 and cRAD51 was half of that observed with cBRC4 and similar to that with hBRC3 and hBRC4. The interaction between hRAD51 and cBRC3 was stronger than with hBRC3. (A) VP16 transactivation domain-fused cRAD51 construct or empty vector and a Gal4-DBD-fused cBRC3, cBRC4, hBRC3, hBRC4, or cBRCA2 N-terminus (1–1000 aa) were introduced into HeLa cells to determine their interaction with cRAD51 in a mammalian two-hybrid luciferase-based assay. HeLa cells were co-transfected with the BRC repeat constructs and cRAD51 expression vector constructs with the reporter plasmids pGluc and pRL-TK. The pRL-TK construct was used to normalize the transfection efficiency. Lysate luciferase activity was determined 48 h after transfection. (B) same as (A), except that VP16 transactivation domain-fused hRAD51 was used. The results are given as the mean (± standard error) (n = 3). Significance was examined by a one-way analysis of variance test, followed by a Tukey's post-test. Different letters indicate significant differences between the cells transfected with the indicated constructs. (p<0.01). DBD, DNA-binding domain; TAD, transactivation domain-fused proteins.

In a previous study, cBRC3 was shown to interact weakly with cRAD51, and this level of interaction was approximately 10% of the extent of the interaction of cRAD51 with cBRC4 [16]. This interaction was repeated during this study using a mammalian two-hybrid assay. Unexpectedly, the level of interaction between cBRC3 and cRAD51 was strong, about half of that of the cBRC4–cRAD51 interaction and similar to that of hBRC3 and hBRC4, which has already been reported to interact strongly with RAD51 [15], [25] (Figure 1 A). The level of interaction between cBRC3 and hRAD51 was also shown to be stronger than that between hBRC3 and hRAD51 (Figure 1 B). This assay also showed that the level of interaction between hBRC3, cBRC4 or hBRC4, and cRAD51 or hRAD51 was different, depending on the animal species of RAD51 (Supporting information Figure S1).

### T1425P mutation disrupts the *in silico* conformation of cBRC3 and RAD51

**Figure 2:**
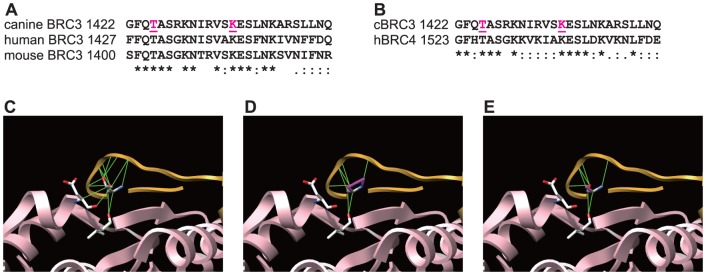
The T1425P mutation disrupted the conformation of canine BRC3 and RAD51 *in silico.* (A) Amino acid sequence alignment of BRC3 among canines (Accession No. NP_001006654.2), humans (Accession No. NP_000050.2), and mice (Accession No. NP_033895.2). (B) Amino acid sequence alignment of cBRC3 and hBRC4. The pink characters indicate the positions of missense mutations in cBRC3. (C–E) The contacts between the residues of 1526T (C), T1526P (D), or T1526A (E) and hRAD51 or other residues of BRC4 were calculated and are depicted. Human BRC4 and hRAD51 are depicted by gold and pink ribbons, respectively. Solid green lines signify stable contacts as determined by the University of California, San Francisco Chimera software.

To verify the effect of the 2 previously reported missense mutations in cBRC3, derived from mammary tumor samples, the mutation tool in the UCSF Chimera software package was used to analyze the possible structural outcomes of these substitutions [19]. This software requires the 3D structure of target proteins or peptides as an input. However, no studies have reported the 3D structure of the complex between BRC repeats and RAD51, excluding that of the complex between hBRC4 and hRAD51. Assuming that cBRC3's interactions with RAD51 were similar to that involving hBRC4, the 3D structure of the complex between hBRC4 and hRAD51 was used as the software input. This seemed rational as both the amino acid sequences and the binding intensities of cBRC3 and hBRC4 with hRAD51 were similar (Figures 1 and 2 B). The rotamers tool allowed amino acid sidechain rotamers to be viewed and evaluated [23]. The best rotamers of T1526P, homologous to the T1425P mutation in cBRC3, were selected, according to their sidechain torsion and probability values in the rotamer library and in the context of the structural environment (Figure 2 D). The calculations revealed that the substitution of T1526P decreased the number of contacts for all kinds of direct interactions. 1526T showed 4 contacts with 187V and 189V of RAD51 and 9 contacts with other residues of hBRC4, while 1526P showed only 2 contacts with 189V of RAD51 and 5 contacts with other residues of hBRC4. The effects of T1526A substitutions on hBRC4, which disrupts the β-hairpin structure and interaction with hRAD51, were also verified [26]. The T1526A substitution showed the same results as the T1526P substitution. However, no detectable effect of the K1536R substitution was identified. The K1536R substitution occurs in a site homologous to the K1435R mutation in cBRC3, on the 3D structure of the hBRC4 and hRAD51 complex.

### The T1425P mutation does not allow interaction with canine and human RAD51 *in vitro* and *in vivo*

**Figure 3:**
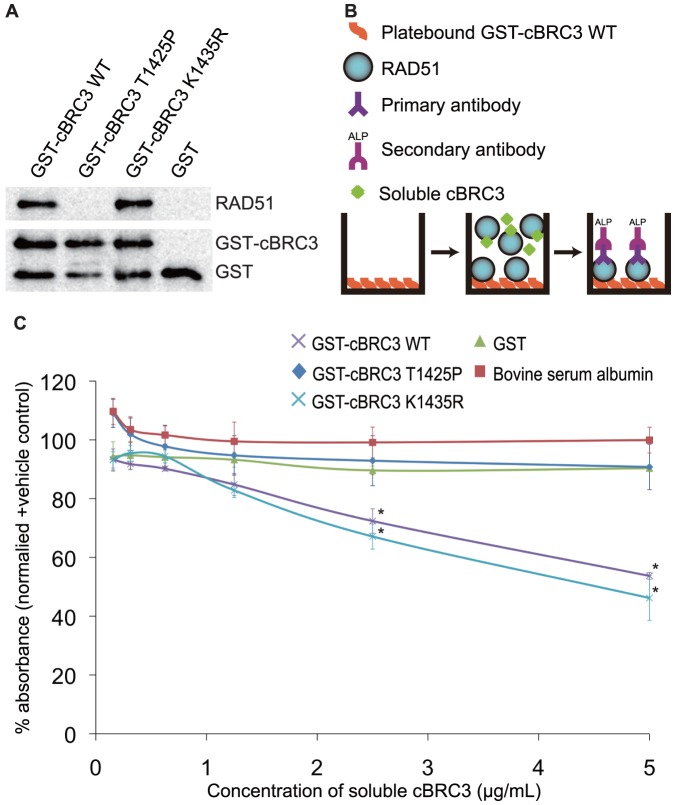
Effect of mutations in canine BRC3 on the interaction with human RAD51 detected *in vitro*. (A) The GST pull-down assay showed that wild-type and K1435R mutant canine BRC3 (cBRC3) interacted with RAD51, but the T1425P mutant cBRC3 did not interact with RAD51. GST-fused peptides were incubated with human RAD51 (hRAD51) at 4°C for 1 h. Equilibrated beads were incubated with the protein–peptide complexes at 4°C for 1 h. Complexes bound to the beads were thoroughly washed. The samples were analyzed by SDS-PAGE and western blotting with anti-RAD51 IgG, or anti-GST IgG, and horseradish peroxidase conjugated anti-rabbit IgG. Blots were developed using the ECL Plus Western Blotting Detection System. (B) Schematic of the enzyme-linked immunosorbent assay (ELISA) used to detect the cBRC-hRAD51 interaction and its disruption by soluble peptides. (C) In a competitive ELISA, GST-cBRC3 with the T1425P mutation did not inhibit the solid-phase interaction between GST-cBRC3 and hRAD51. However, the substitution of GST-cBRC3 with the K1435R mutation showed no interaction with hRAD51, like wild-type cBRC3. Values are expressed as the mean percentage absorbance at 405 nm for data sets in triplicate normalized to the positive control, which was not treated with the soluble inhibitors, and the negative control, which was coated with GST and treated with hRAD51. Significance was examined by a one-way analysis of variance test followed by Tukey's post-test. The asterisks indicate a significant difference compared with GST-treated data sets at the same competitor concentration (p<0.01).

The interaction between mutant cBRC3 and hRAD51, was examined using a pull-down assay and competitive ELISA (Figure 3). GST-fused mutant cBRC3 peptides were purified and used to perform a GST pull-down assay. As expected, the T1425P mutant cBRC3 did not interact with hRAD51, while wild-type cBRC3 and the K1435R mutant of cBRC3 did interact with hRAD51 (Figure 3 A). To quantify the interaction between mutant cBRC3 and hRAD51, a competitive ELISA was performed (Figure 3 B and C). Wild-type and K1435R mutant cBRC3 peptides significantly competed for the interaction between wild-type cBRC3 and hRAD51, compared with that between GST or BSA and hRAD51. However, the T1425P mutation had no effect on the interaction with hRAD51. Thus, the results of the GST pull-down assay and competitive ELISA indicated that the interaction of cBRC3 with hRAD51 was almost entirely abolished by the T1425P mutation.

**Figure 4:**
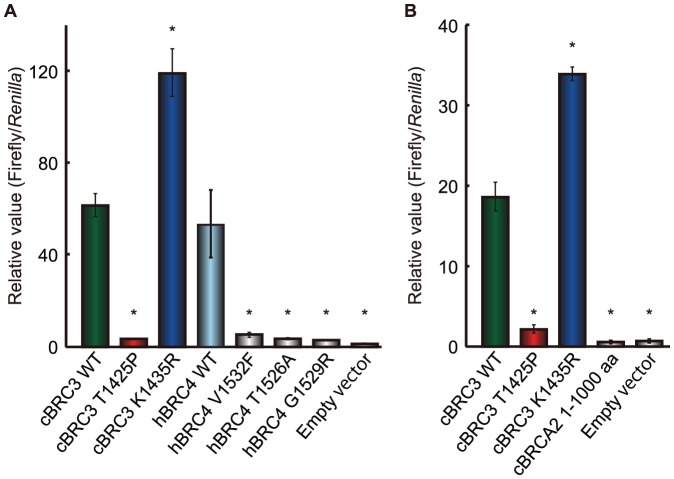
Effect of mutations in canine BRC3 on its interaction with canine or human RAD51 *in vivo.* The binding intensity of T1425P mutant canine BRC3 (cBRC3) and canine RAD51 (cRAD51) or human RAD51 (hRAD51) was much lower than that of wild-type cBRC3 and cRAD51 or hRAD51, while that of the K1435R mutant cBRC3 and cRAD51 or hRAD51 was higher than between wild-type cBRC3 and cRAD51 or hRAD51. (A) Gal4-DNA binding domain-fused wild-type and mutant cBRC3 or mutants of human BRC4 (hBRC4) and VP16 transactivation domain-fused cRAD51 constructs were introduced into HeLa cells to determine their interaction with cRAD51 in a mammalian two-hybrid assay measuring luciferase activity. HeLa cells were co-transfected with the BRC repeat constructs and cRAD51 expression vector constructs and with the reporter plasmids pGluc and pRL-TK. The pRL-TK construct was used to normalize the transfection efficiency. Lysate luciferase activity was determined 48 h after transfection. (B) same as (A), except that VP16 transactivation domain-fused hRAD51 was used. The results are given as the mean (± standard error) (n = 3). Significance was examined by a one-way analysis of variance test followed by Dunnett's post-test. The asterisks indicate a significant difference compared with cells transfected with wild-type (WT) cBRC3 (p<0.01).

To reconfirm the interaction between cBRC3 mutants and RAD51, each mutant and wild-type cBRC3 was compared using a mammalian two-hybrid assay involving HeLa cells (Figure 4). The binding intensity of the T1425P mutant cBRC3 and cRAD51 or hRAD51 was much lower than that of wild-type cBRC3 and cRAD51 or hRAD51; it was equivalent to that of V1532F, T1526A, and G1529R mutant human BRC4, which were reported to entirely abolish the interaction with RAD51, and the cBRCA2 N-terminus, which does not have any interaction domains with RAD51 [15], [27]. A stronger interaction between the K1435R mutant cBRC3 and cRAD51 or hRAD51 was observed than between wild-type cBRC3 and cRAD51 or hRAD51.

### T1425P mutant cBRC3 does not inhibit RAD51 self-association

**Figure 5:**
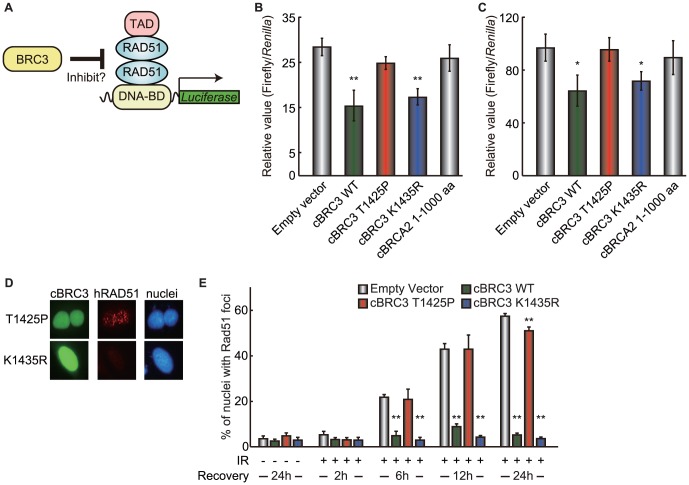
The T1425P mutant canine BRC3 does not inhibit canine or human RAD51 self-association. (A) Schematic of the modified mammalian two-hybrid assay used to examine how mutant canine BRC3 (cBRC3) interferes with the RAD51-RAD51 interaction. (B) The graph shows the strength of the interference caused by the expression of EGFP empty vector, wild-type EGFP-cBRC3, 2 mutants of cBRC3 (T1425P and K1435R), and cBRCA2 N-terminus (1–1000 aa) on the canine RAD51 (cRAD51)-cRAD51 interaction as determined by the modified mammalian two-hybrid assay. Significance was examined by a one-way analysis of variance test followed by Dunnett's post-test. The asterisks indicate a significant difference compared with the cells transfected with an empty vector (*: p<0.05, **: p<0.01). The negative control for this assay is shown in Supporting information Figure S2. (C) same as (B), except that human RAD51 (hRAD51) was used. (D) Immunostained cells, which were transiently transfected with cBRC3 mutants, after being subjected to ionizing radiation, are shown. The indicated cells were irradiated (15 Gy) and fixed 12 h after exposure to the ionizing radiation. (E) HeLa cells transfected with wild-type or mutant cBRC3 or empty vector were irradiated (15 Gy) and then allowed to recover for the times indicated. Images containing at least 100 cells were captured by a computer, and the number of cells containing at least 10 foci were recorded and plotted as a percentage of the total number of cells. The plots were generated from 3 independent experiments. The results are given as the mean (± standard error) (n = 3). Significance was examined by a one-way analysis of variance test followed by Dunnett's post-test. The asterisks indicate a significant difference compared with irradiated cells transfected with an empty vector at the same time point (p<0.01).

BRC repeats, including BRC3, mimic a motif in RAD51 that functions as an interface for oligomerization between individual RAD51 monomers, and they inhibit RAD51 self-association and RAD51 nucleofilament formation [26], [28], [29]. To measure the extent to which each cBRC3 mutation interferes with RAD51 self-association, a modified mammalian two-hybrid assay was performed (Figure 5 and Supporting information Figure S2). The T1425P mutant cBRC3 did not inhibit cRAD51 or hRAD51 self-association, although wild-type and the K1435R mutant cBRC3 did inhibit this association (Figure 5 B and C).

To confirm the results of the modified mammalian two-hybrid assay, the effect of the cBRC3 mutants on endogenous hRAD51 oligomerization was investigated using a RAD51 focus formation assay. The RAD51 foci were slightly affected by transfection with the T1425P mutant cBRC3, although their number was reduced by transfection with wild-type or K1435R mutant cBRC3 (Figure 5 D and E). This assay also showed that the inhibition of RAD51 foci formation by the K1435R mutant cBRC3 was stronger than that of the wild-type cBRC3 in X-ray-exposed cells after 12 and 24 h (Supporting information Figure S3).

### Effect of substitutions similar to the cBRC3 mutations on hBRC3

**Figure 6:**
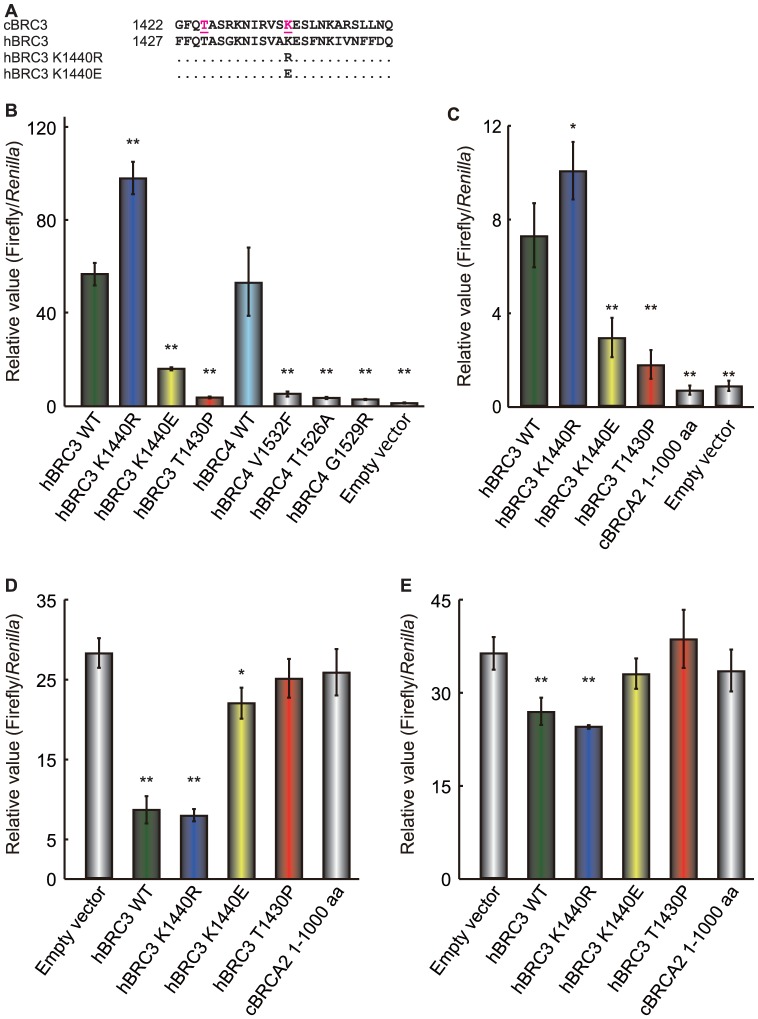
Effect of mutations in human BRC3 on its interaction with canine or human RAD51 *in vivo.* (A) Amino acid sequence alignment among canine BRC3 (cBRC3) (Accession No. NP_001006654.2), wild-type human BRC3 (hBRC3) (Accession No. NP_000050.2), and 2 mutants of hBRC3. The pink characters indicate the positions of missense mutations in cBRC3. (B) Gal4-DNA binding domain-fused wild-type and mutant hBRC3 and VP16 transactivation domain-fused cRAD51 constructs were introduced into HeLa cells to determine their interaction with cRAD51 in a mammalian two-hybrid assay measuring luciferase activity. HeLa cells were co-transfected with the hBRC3 constructs and canine RAD51 (cRAD51) expression vector constructs and with the reporter plasmids pGluc and pRL-TK. The pRL-TK construct was used to normalize the transfection efficiency. Lysate luciferase activity was determined 48 h after transfection. The results are given as the mean (± standard error) (n = 3). Significance was examined by a one-way analysis of variance test followed by Dunnett's post-test. The asterisks indicate a significant difference compared to cells transfected with wild-type (WT) cBRC3 (*: p<0.05, **: p<0.01). (C) same as (B), except that VP16 transactivation domain-fused hRAD51 was used. (D) The graph shows the strength of the interference caused by the expression of EGFP empty vector, wild-type EGFP-hBRC3, 3 mutants of hBRC3 (K1440R, K1440E, and T1430P), and cBRCA2 N-terminus (1–1000 aa) on the cRAD51-cRAD51 interaction as determined by the modified mammalian two-hybrid assay. Significance was examined by one-way ANOVA test followed by Dunnett's test. The asterisks indicate a significant difference compared to the cells transfected with an empty vector (*: p<0.05, **: p<0.01). The negative control for this assay is shown in Supporting information Figure S2. (E) same as (D), except that hRAD51 was used.

The T1425P and K1435R mutations, discovered in canine mammary tumor samples, affected the interaction of cBRC3 with both cRAD51 and hRAD51. Mutations (K1440R and K1440E) analogous to the K1435R mutation in cBRC3 were reported in human breast cancer patients in the Breast Cancer Information Core (http://research.nhgri.nih.gov/projects/bic/index.shtml). Therefore, an attempt was made to extrapolate the findings regarding cBRCA2 to hBRCA2. To evaluate the importance of the K1440R and K1440E human breast cancer mutations and the T1430P substitution mutation in hBRC3, a mammalian two-hybrid assay was performed using HeLa cells (Figure 6). The binding intensity of the K1440R mutant hBRC3 to cRAD51 or hRAD51 was stronger than that of wild-type hBRC3 (Figure 6 B and C). In contrast, the binding intensity of the T1430P mutant hBRC3 to cRAD51 or hRAD51 was much lower than that of the wild-type hBRC3; in fact, it was equivalent to the binding associated with the V1532F, T1526A, and G1529R mutants of hBRC4, and the N-terminus of the cBRCA2. The hBRC3's ability to inhibit human or canine RAD51 self-association also disappeared as a result of the T1430P substitution, but the K1440R mutant hBRC3 still possessed that inhibitory activity (Figure 6 D and E). These results corresponded to the findings with cBRC3. The interaction of the K1440E mutant hBRC3 with cRAD51 or hRAD51 was weaker than that of wild-type hBRC3 (Figure 6 B and C). In accordance with this finding, the ability of hBRC3 to inhibit RAD51 self-association was only slightly affected by the K1440E mutation, and this effect was only observed when the effect of the K1440E mutant was tested on cRAD51 self-association (Figure 6 D).

## Discussion

In this study, the effect of 2 missense mutations located in cBRC3, an amino acid sequence that is important for mediating the interaction between BRCA2 and RAD51 [13], [19], was examined for their effect on the functioning of cBRC3. By assuming that the tertiary structure of cBRC3 and its resultant interactions with hRAD51 were similar to those of hBRC4, the UCSF Chimera software predicted that the T1425P mutation affects the interaction between cBRC3 and RAD51. The effect was predicted to be similar to that caused by the T1526A mutation, which disrupts the hBRC4 structure and its interaction with hRAD51. The results from the pull-down assay, the comparative ELISA, and the mammalian two-hybrid assay indicated that the presence of the T1425P mutation almost entirely abolished the interaction between cBRC3 and cRAD51 or hRAD51.

BRC repeats also mimic the motif in RAD51 responsible for RAD51 self-association and, thereby, inhibit this association [26], [28], [29]. The effect of the T1425P mutation on the inhibition of cRAD51 or hRAD51 self-association, using a modified mammalian two-hybrid assay and a RAD51 focus formation assay, showed that the T1425P mutant cBRC3 did not inhibit cRAD51 or hRAD51 self-association. These results suggested that the T1425P mutant cBRC3 does not function in the molecular mimicry-based inhibition of RAD51 self-association. Previous structural analyses of cancer-associated mutations affecting BRC repeats showed that the weakening of RAD51 affinity in the case of even 1 repeat is adequate to increase breast cancer susceptibility [26], [30], [31]. Therefore, the T1425P mutation might be similarly related to canine mammary tumors.

Another mutation in cBRC3, K1435R, functioned similarly to wild-type cBRC3, except in the mammalian two-hybrid assay and RAD51 focus formation assay where the mutant showed stronger interactions with cRAD51 and hRAD51, inhibiting RAD51 self-association to a greater extent than the wild-type cBRC3. However, there were no apparent differences between the K1435R mutant and wild-type cBRC3 in the other analyses. Further, the same mutation (K1440R) in hBRC3, detected in human breast cancer patients, also showed stronger interactions with cRAD51 and hRAD51 than the wild-type hBRC3. These results led to the inference that that none of the analyses used in this study, except the mammalian two-hybrid assay and RAD51 focus formation assay, could detect this difference because they were unable to detect such slight differences. In both dogs and humans, these mutations might be involved in mammary tumors as a result of their effect on the regulation of RAD51 recruitment. However, this hypothesis requires further investigation.

The T1430P and K1440R hBRC3 mutants, corresponding to the T1425P and K1435R mutants in canine BRCA2, yielded results similar to those for the cBRC3 mutants, i.e., the interaction between hBRC3 and cRAD51 or hRAD51 was almost entirely abolished by the T1430P mutation and induced by the K1440R mutation. These results further support the concept that the structure and important amino acids are similar between canine and human BRC3s. Although further confirmation is required to show the similarity between canine and human BRCA2 functions, cBRCA2 is speculated to have a function similar to that of hBRCA2 in mammary tumors and may be a useful model of human breast cancer resulting from BRCA2 mutations.

In this study, the K1440E mutation, found in the hBRC3 of human breast cancer patients, reduced the interaction of the mutant protein with cRAD51 and hRAD51 and reduced or abolished the ability of RAD51 to self-associate. Although the specific risk of a K1440E mutation has not been investigated, previous structural analyses of cancer-associated mutations associated with the BRC repeats strongly indicated that weakened RAD51 affinity in the case of even 1 BRC repeat is adequate to increase breast cancer susceptibility [26], [30], [31]. Therefore, the K1440E mutation might be related to human breast cancer. In contrast to K1440E, the K1440R mutation strengthened the interaction between cRAD51 or hRAD51 and hBRC3. These data demonstrate the importance of 1440K in BRC repeats in terms of their interactions with RAD51. Although previous reports have identified some of the BRC repeat amino acids that are important for mediating the interaction with RAD51 [28], [32], [33], this amino acid has not been previously identified. Thus, the present study appears to be the first to reveal the importance of the 1440K amino acid. Further studies are, however, required to clarify the effect of these mutations on BRCA2 function.

This study also demonstrated that cBRC3 strongly interacts with cRAD51, to an extent similar to hBRC3 and hBRC4. A previous publication reported that cBRC3 interacted weakly with cRAD51 [16]. One potential reason for this weak interaction may be that the BRC3 region used in the 2 studies was different; in one, the amino acid sequence 1410–1482 was used, whereas in the other, the amino acid sequence 1397–1502 was tested. Thus, amino acids 1397–1409 and 1483–1502 may exhibit a suppressive function towards the interaction with cRAD51. The interaction between canine or human BRC3 or BRC4 and canine or human RAD51 differed, depending on the animal species of RAD51. The level of interaction between hBRC3 with cRAD51 was markedly stronger than it was with hRAD51. There are 3 amino acids that differ between hRAD51 and cRAD51, leading to the speculation that these amino acids affect the interaction between BRC repeats and RAD51.

In conclusion, 2 canine mammary tumor missense mutations (T1425P and K1435R) in cBRC3 and 2 human breast cancer mutations (K1440R and K1440E) in hBRC3 affect BRC3 function and, therefore, may be associated with canine and human mammary tumors. Further, hBRC3 mutations (T1430P and K1440R) were shown to correspond to the T1425P and K1435R mutations in canine BRCA2 and yield similar results. Therefore, we conclude that canine BRCA2 may be a good model for studying human breast cancer caused by human BRCA2 mutations.

## Supporting Information

Figure S1 **Interaction between canine and human BRC3 or BRC4 and canine or human RAD51.** (A) The VP16 transactivation domain-fused cRAD51 construct or empty vector and Gal4-DBD-fused cBRC3, cBRC4, hBRC3, or hBRC4 were introduced into HeLa cells to characterize their interaction with cRAD51 or hRAD51 in a mammalian two-hybrid assay measuring luciferase activity. HeLa cells were co-transfected with the BRC repeat constructs and RAD51 expression vector constructs with the reporter plasmids pGluc and pRL-TK. The pRL-TK construct was used to normalize transfection efficiency. Lysate luciferase activity was determined 48 h after transfection. Results are given as the mean (± standard error) (n = 3). Significance was determined by student's *t*-test. Asterisks indicate significant difference between cells transfected with cRAD51 and those with hRAD51. DBD and TAD indicate DNA binding domain-fused proteins and transactivation domain-fused proteins, respectively.
(PDF)

Figure S2 **Negative control for the modified mammalian two-hybrid assay.** The graph shows the negative control for the modified mammalian two-hybrid assay (Figures 5 A–C, 6 D and E). Cells were transfected with the DNA binding domain (DBD)-fused canine or human RAD51 and EGFP-fused canine or human BRC3, or the cBRCA2 N-terminus (1–1000 aa) with or without the transactivation domain (TAD)-fused canine or human RAD51.
(PDF)

Figure S3 **Inhibition of hRAD51 foci formation of K1435R mutant cBRC3 versus wild-type cBRC3.** HeLa cells transfected with wild-type or mutant cBRC3 or empty vector were irradiated (15 Gy) and then allowed to recover for the times indicated. Images containing at least 100 cells were captured by a computer, and the number of cells containing at least 10 foci were recorded and plotted as a percentage of the total number of cells. Plots were generated from 3 independent experiments. The results are given as the mean (± standard error) (n = 3). Significance was examined by student's *t*-test. Asterisks indicate significant difference between irradiated cells transfected with wild-type and with K1435R mutant cBRC3 at the same time point (*p<0.05, **p<0.01).
(PDF)
