# Supplementary material for: Effects of the Missense Mutations in Canine BRCA2 on BRC Repeat 3 Functions and Comparative Analyses between Canine and Human BRC Repeat 3
Source: PLoS One. 2012 Oct 12;7(10):e45833. doi: 10.1371/journal.pone.0045833 (PMC3470543; doi:10.1371/journal.pone.0045833)
Supplement: Figure S1 — Interaction between canine and human BRC3 or BRC4 and canine or human RAD51. (A) The VP16 transactivation domain-fused cRAD51 construct or empty vector and Gal4-DBD-fused cBRC3, cBRC4, hBRC3, or hBRC4 were introduced into HeLa cells to characterize their interaction with cRAD51 or hRAD51 in a mammalian two-hybrid assay measuring luciferase activity. HeLa cells were co-transfected with the BRC repeat constructs and RAD51 expression vector constructs with the reporter plasmids pGluc and pRL-TK. The pRL-TK construct was used to normalize transfection efficiency. Lysate luciferase activity was determined 48 h after transfection. Results are given as the mean (± standard error) (n = 3). Significance was determined by student's t-test. Asterisks indicate significant difference between cells transfected with cRAD51 and those with hRAD51. DBD and TAD indicate DNA binding domain-fused proteins and transactivation domain-fused proteins, respectively. (PDF) [file pone.0045833.s001.pdf]

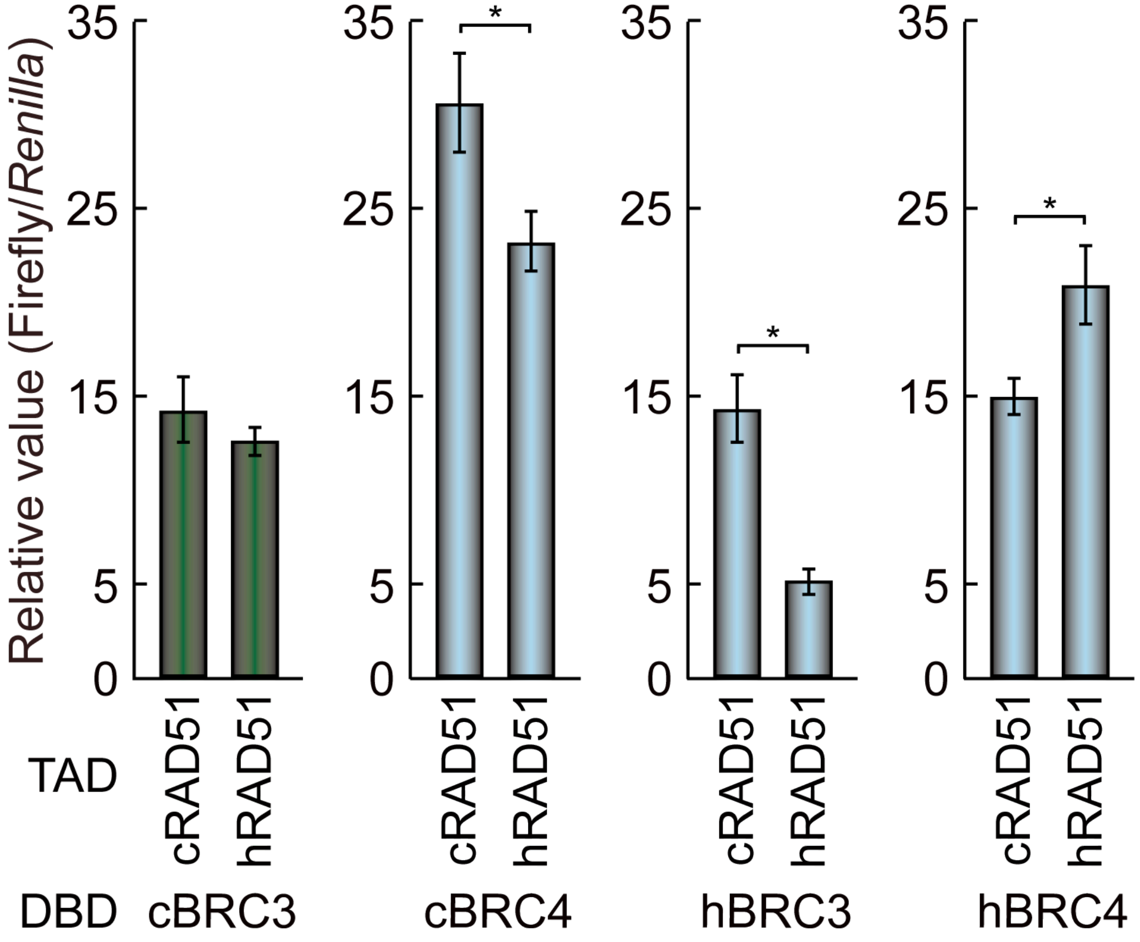

Supporting information Figure S1. Interaction between canine and human BRC3 or BRC4 and canine or human RAD51

(A) The VP16 transactivation domain-fused cRAD51 construct or empty vector and Gal4-DBD-fused cBRC3, cBRC4, hBRC3, or hBRC4 were introduced into HeLa cells to characterize their interaction with cRAD51 or hRAD51 in a mammalian two-hybrid assay measuring luciferase activity. HeLa cells were co-transfected with the BRC repeat constructs and RAD51 expression vector constructs with the reporter plasmids pGluc and pRL-TK. The pRL-TK construct was used to normalize transfection efficiency. Lysate luciferase activity was determined 48 h after transfection.

Results are given as the mean (± standard error) (n = 3). Significance was determined by student's *t*-test. Asterisks indicate significant difference between cells transfected with cRAD51 and those with hRAD51. DBD and TAD indicate DNA binding domain-fused proteins and transactivation domain-fused proteins, respectively.
