# Supplementary material for: Effects of the Missense Mutations in Canine BRCA2 on BRC Repeat 3 Functions and Comparative Analyses between Canine and Human BRC Repeat 3
Source: PLoS One. 2012 Oct 12;7(10):e45833. doi: 10.1371/journal.pone.0045833 (PMC3470543; doi:10.1371/journal.pone.0045833)
Supplement: Figure S2 — Negative control for the modified mammalian two-hybrid assay. The graph shows the negative control for the modified mammalian two-hybrid assay (Figures 5 A–C, 6 D and E). Cells were transfected with the DNA binding domain (DBD)-fused canine or human RAD51 and EGFP-fused canine or human BRC3, or the cBRCA2 N-terminus (1–1000 aa) with or without the transactivation domain (TAD)-fused canine or human RAD51. (PDF) [file pone.0045833.s002.pdf]

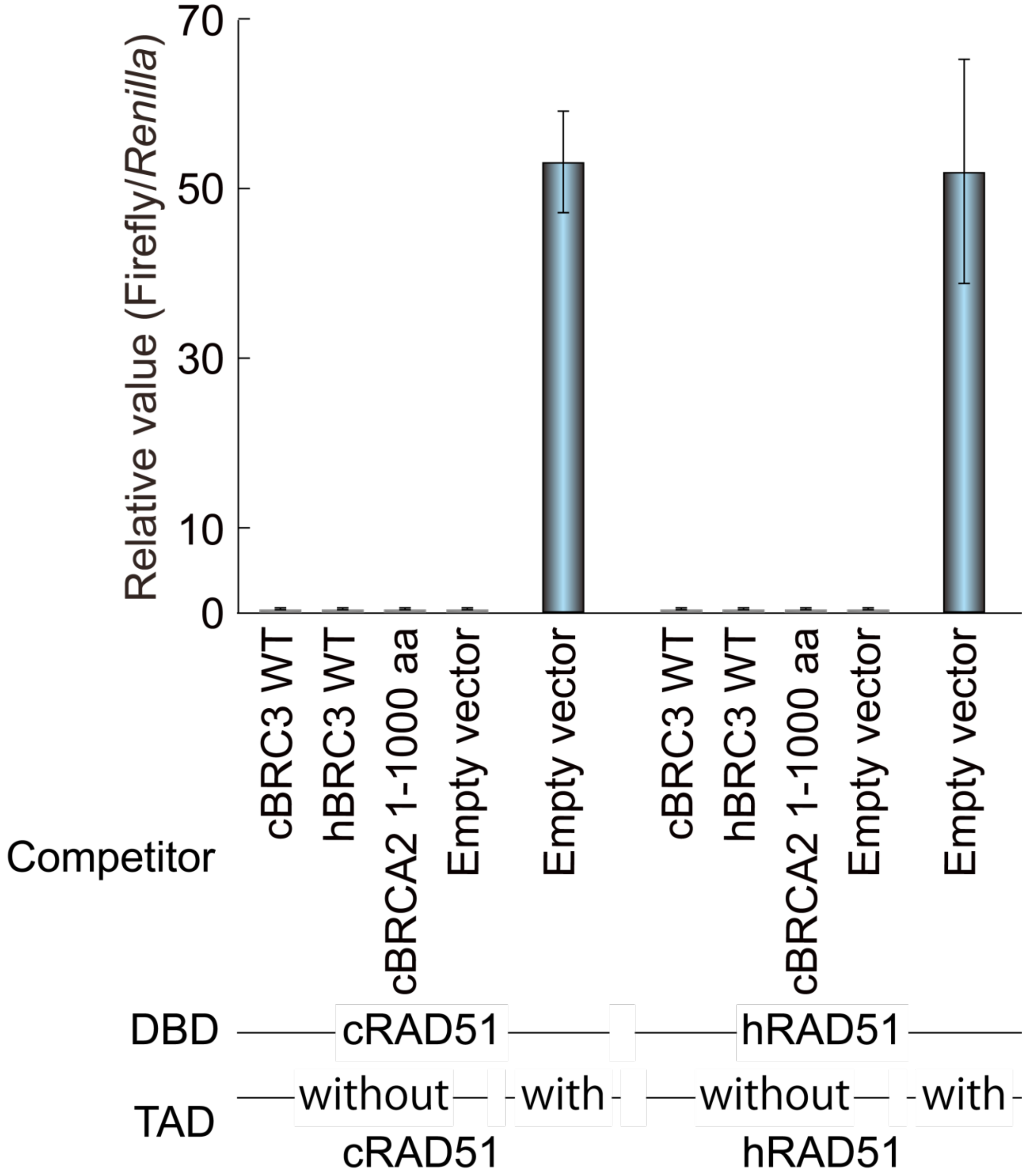

Supporting information Figure S2. Negative control for the modified mammalian two-hybrid assay

The graph shows the negative control for the modified mammalian two-hybrid assay (Figures 5 A-C, 6 D and E). Cells were transfected with the DNA binding domain (DBD)-fused canine or human RAD51 and EGFP-fused canine or human BRC3, or the cBRCA2 N-terminus (1–1000 aa) with or without the transactivation domain (TAD)-fused canine or human RAD51.
