# Supplementary material for: Effects of the Missense Mutations in Canine BRCA2 on BRC Repeat 3 Functions and Comparative Analyses between Canine and Human BRC Repeat 3
Source: PLoS One. 2012 Oct 12;7(10):e45833. doi: 10.1371/journal.pone.0045833 (PMC3470543; doi:10.1371/journal.pone.0045833)
Supplement: Figure S3 — Inhibition of hRAD51 foci formation of K1435R mutant cBRC3 versus wild-type cBRC3. HeLa cells transfected with wild-type or mutant cBRC3 or empty vector were irradiated (15 Gy) and then allowed to recover for the times indicated. Images containing at least 100 cells were captured by a computer, and the number of cells containing at least 10 foci were recorded and plotted as a percentage of the total number of cells. Plots were generated from 3 independent experiments. The results are given as the mean (± standard error) (n = 3). Significance was examined by student's t-test. Asterisks indicate significant difference between irradiated cells transfected with wild-type and with K1435R mutant cBRC3 at the same time point (*p<0.05, **p<0.01). (PDF) [file pone.0045833.s003.pdf]

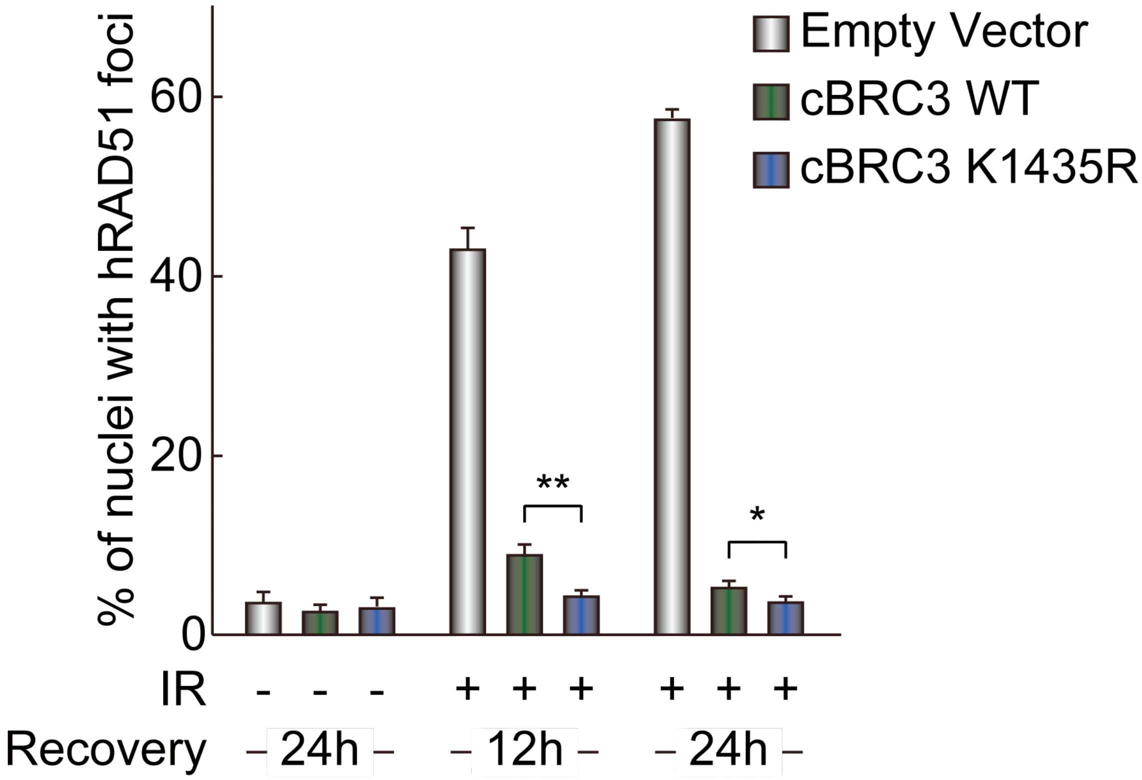


**Supporting information Figure S3. Inhibition of hRAD51 foci formation of K1435R mutant cBRC3 versus wild-type cBRC3**

HeLa cells transfected with wild-type or mutant cBRC3 or empty vector were irradiated (15 Gy) and then allowed to recover for the times indicated. Images containing at least 100 cells were captured by a computer, and the number of cells containing at least 10 foci were recorded and plotted as a percentage of the total number of cells. Plots were generated from 3 independent experiments. The results are given as the mean (± standard error) ($n = 3$). Significance was examined by student's *t*-test. Asterisks indicate significant difference between irradiated cells transfected with wild-type and with K1435R mutant cBRC3 at the same time point (*$p < 0.05$, **$p < 0.01$).
